# The mediating role of sleep problems and depressed mood between psychological abuse/neglect and suicidal ideation in adolescent childhood: a multicentred, large sample survey in Western China

**DOI:** 10.1186/s12888-024-05503-x

**Published:** 2024-01-23

**Authors:** Yu Cen, Jinlong He, Yunling Zhong, Jinhui Zhou, Jiaxin Zeng, Guoping Huang, Jiaming Luo

**Affiliations:** 1 Mental Health Center, Affiliated Hospital of North Sichuan Medical College, Nanchong, China; 2 School of Psychiatry, North Sichuan Medical College, Nanchong, China; 3 Department of Psychiatry, Nanchong Psychosomatic Hospital, Nanchong, China

**Keywords:** Psychological abuse, Psychological neglect, Sleep problems, Depressed mood, Suicidal ideation, Mental health, Childhood maltreatment, Adolescent

## Abstract

**Background:**

Adolescent suicidal ideation are associated with factors including psychological abuse/neglect, sleep problems, and depressed mood, but the systematic effects of these factors on suicidal ideation remain unclear, which is a research gap this work aims to fill.

**Methods:**

A multi-center, the cluster sampling method was employed to collect general demographic data, such as age, gender, the experience of being left behind, and parents’ marital status, from 12,192 students across 17 secondary schools in China. The Child Psychological Abuse and Neglect Scale (CPANS), Pittsburgh Sleep Quality Index (PSQI), the Chinese version of the Depressed mood, Anxiety and Stress Scale − 21 Items (DASS-21) and Chinese version of Positive and Negative Suicide Ideation Inventory (PANSI) were utilized. Data were analyzed using t-tests, chi-square tests, correlation analyses, and structural equation modeling mediation analyses.

**Results:**

The prevalence of psychological abuse/neglect and adolescent suicidal ideation was 34.8% and 13%, respectively. This mediation analysis suggests that, in the relationship between psychological abuse/neglect and suicidal ideation, sleep problems and depressed mood play both parallel and sequential mediating roles.

**Conclusion:**

Sleep problems and depressed mood play a mediating role in the development of suicidal ideation in adolescents. Good sleep habits and depressed mood interventions help reduce the risk of suicidal ideation in adolescents who experience psychological neglect/abuse.

## Introduction

Suicide has emerged as a substantial public health concern, becoming the third leading cause of death among 15-19-year-olds worldwide. Over 700,000 individuals die by suicide each year, taking a heavy toll on individuals, families and even society [1]. Adolescents, who undergo rapid physical, emotional, and environmental changes, are particularly vulnerable and may resort to extreme coping mechanisms when faced with negative events or challenges. In China, although the overall suicide rate has experienced a significant decline in recent years, the prevalence of youth suicide remains high and demands urgent attention [2]. Suicidal ideation is a direct precursor to suicide attempts and is critical for predicting and preventing suicidal behavior through early intervention [3–5]. Thus, it is imperative to investigate the risk factors associated with suicidal ideation and implement appropriate measures to mitigate and prevent suicidal behavior among adolescents.

Psychological abuse/neglect, the core issues of child abuse, involve persistent and inappropriate behavior by a child’s guardian or parent that is predominantly emotional, such as neglecting the child’s needs, indifference, derogation, and intimidation, without involving physical or sexual contact [6]. Compared to physical and sexual abuse, psychological abuse/neglect is insidious, difficult to identify and measure, highly prevalent and currently under-researched [7]. Although it is common in all countries, Chinese parents may generally exhibit less acceptance, consistency, enthusiasm, and more restrictive, hostile, rejecting, or neglectful behaviors than parents from other cultures [8]. This makes psychological abuse/neglect a potentially important public health issue in China. Previous studies have demonstrated that psychological abuse/neglect can adversely affect adolescent development, and may even contribute to the emergence and progression of suicidal ideation [9, 10, 11, 12–14]. For example, Miller et al [10]. showed through a three-year prospective study that emotional abuse was a strong predictor of suicidal ideation. The stress-quality theory of suicide emphasizes that the interaction between early traumatic events and biologically based susceptibility qualities increases the likelihood of suicidal behaviour, this also indicates that psychological abuse/neglect can exacerbate the vulnerability to suicide in adolescents experiencing a tumultuous developmental phase [11]. Previous studies have shown a link between child psychological abuse/neglect and suicidal ideation, but how this psychological abuse/neglect affects adolescent suicidal ideation over time and by what mechanisms is unclear. This study aims to further investigate these mechanisms.

Sleep problems are common among teenagers and the most common problems include insomnia, nightmares, and shortened sleep duration [12, 13]. In Japan, 21.1-38.8% of adolescents reported sleep disorders. In Shanghai, China, sleep problems are also prevalent, with 9.2% of junior high school students having poor sleep quality and 84.8% reporting insufficient sleep time [14]. Recent meta-analysis results show that the sleep deficiency rate among Chinese children and adolescents is alarmingly high, reaching 61% [15]. Previous studies have demonstrated that adolescents exposed to child maltreatment are at increased risk for sleep problems, and there is a significant dose-response relationship between cumulative child maltreatment and adolescent sleep disturbances [13, 16]. In addition, studies have found that sleep disturbance can increase suicidal behaviour by 1.95–2.95 times [17]. There was a significant relationship between at least one sleep indicator, including general sleep problems, sleep difficulties and suicidal ideation, which predicted suicidal ideation [18]. In a sample of Chinese university students, emotional abuse predicted suicidal ideation and suicidal behaviour mediated by perceived stress and sleep quality [18]. It appears plausible that the effects of psychological abuse/neglect on sleep problems increase the risk of subsequent suicidal behavior. Consequently, this study proposes Hypothesis 1: Sleep problems may mediate the relationship between psychological abuse/neglect and suicidal ideation.

Depressed mood is a widely studied negative emotion, and research has shown that psychological abuse/neglect are significant predictor of depressed mood, even after controlling for physical and sexual abuse [19, 20]. This may be because children who experience childhood abuse have difficulty regulating their negative thinking, making them more vulnerable to depressed moods. Additionally, depressed moods have a strong association with suicidal behavior [21, 22]. Among adolescents with depressed mood, experiencing childhood maltreatment and the severity of depressed mood increases the risk of suicidal ideation [23]. The quality-stress theory of suicide suggests that a combination of individual susceptibility and external stressors induces suicide [24]. Childhood psychological abuse/neglect represents such external stressors that may lead individuals to suicidal ideation through negative emotions, given that depressed mood is a susceptible quality. Therefore, we propose hypothesis 2 that depressed mood may mediate the relationship between psychological abuse/neglect and suicidal ideation.

Based on the reasoning above, the meidating roles played by sleep problems and depressed mood have been given, but whether their role is parallel or chained needs further validation. Studies have found that sleep disorders, such as insomnia and nightmares, are risk factors for depressed mood [25]. Low levels of sleep quality and high levels of sleep problems trigger depressive symptoms or exacerbate existing depressive symptoms. Depressed mood, in turn, is a risk factor for suicidal ideation, with increased levels contributing to higher ideation levels [26]. Among all sleep-related mental disorders, depression is the most common. Additionally, depression disorders are recognized as a risk factor for suicide [27, 28]. Inadequate sleep quality diminishes an individual’s capacity to regulate emotions, resulting in heightened experience of negative emotions (e.g., depression).This is also consistent with the stress-diathesis model of suicide [24]. The quality of sleep affects suicidal ideation by acting on depressive susceptibility qualities. Therefore, we propose hypothesis 3 that sleep problems, and depressed mood have chain mediating effects in the pathway from psychological abuse/neglect to adolescent suicidal ideation.

**Fig. 1:**
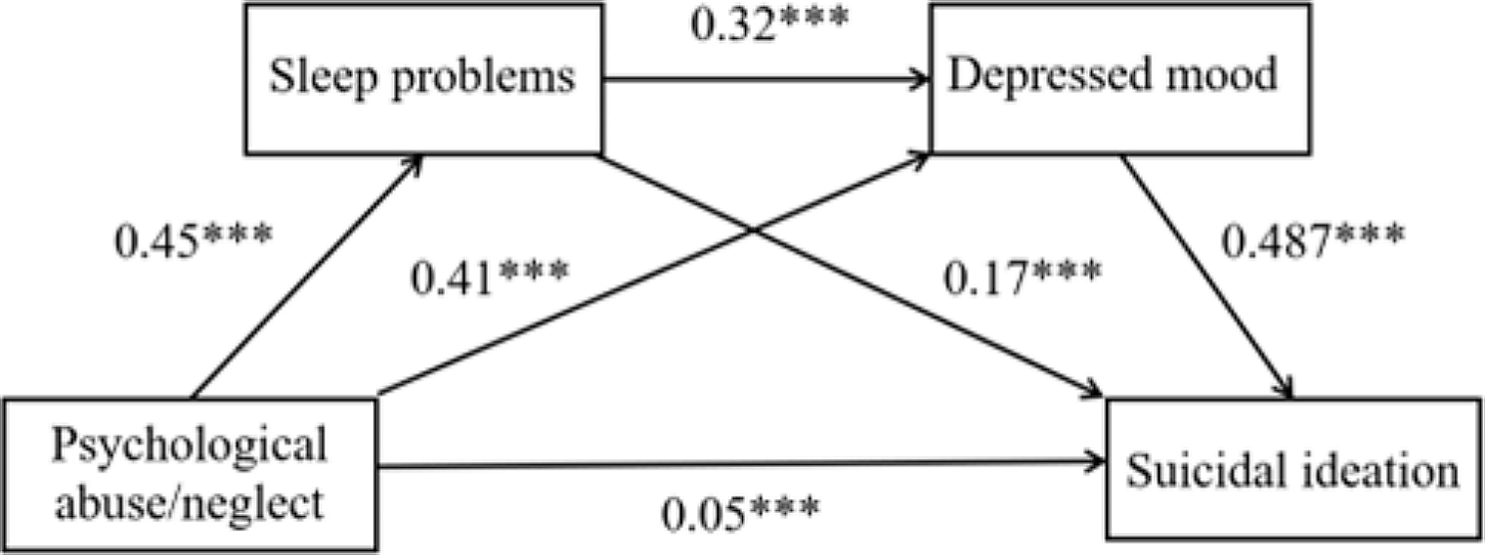
The serial mediation effect Note: Controlled for age, gender, experience of being left behind, parental marital status, being an only child, and long-term medication use; Standardized coefficients are reported; ***p < 0.001

Based on Hypotheses 1, 2, and 3, this study proposes to construct an integrated model (as depicted in Fig. 1) that includes sleep problems and depressed mood to examine the effects of psychological abuse/neglect on adolescent suicidal ideation. To our knowledge, no prior studies have explored this pathway.

## Methods

### Participants and data collection

The research was conducted from November 2021 to May 2022 using a cluster sampling method. Participants were students from 17 secondary schools in Nanchong (15 secondary schools), Neijiang (1 secondary school), and Luzhou (1 secondary school), all of which are located in Sichuan Province. Nanchong, Neijiang, and Luzhou populations were approximately 7.28 million, 4.12 million, and 4.26 million, respectively. In each school, thoroughly trained researchers administered electronic questionnaires to students according to standardized operating procedures (SOPs), and students completed the surveys in class. Students were not allowed to discuss the questionnaire with others, and a time limit was set for completion. Informed consent was obtained from participants prior to the investigation. During the survey, all participants were informed that their privacy would be protected, and they could withdraw at any time if they felt uncomfortable. The study team included psychiatrists, postgraduate students, and undergraduate psychiatry students from the School of Mental Health at North Sichuan Medical University. Each member underwent training in professional knowledge and questionnaire-related content before the survey and passed the relevant tests. Throughout the data collection process, researchers strictly adhered to SOPs and were fully aware of potential issues that might arise during the survey. They had prepared contingency plans for these situations before the study commenced.

This cross-sectional study aimed to evaluate the incidence of suicidal ideation among adolescents. A two-sided test with an αvalue of 0.05 and a tolerance error δ of 0.01 was required. Literature [29] has estimated that 20% of adolescents would experience suicidal ideation. Using PASS15 software, a minimum sample size of 6245 cases was calculated. Considering a 20% data loss or refusal to participate, the minimum sample size was adjusted to 7807.

### Ethics approval

The study received approval from the Ethics Committee of North Sichuan Medical University under project number NSMC [2021] 53 and was reviewed by the Chinese Clinical Trials Registry under registration No.ChiCTR2200058160(24/10/2021).

### Questionnaires

#### Psychological abuse/neglect

The Child psychological abuse/neglect Scale (CPANS) was used to measure psychological abuse/neglect in childhood [6]. The 31-item scale consists of two subscales, i.e., *Psychological Abuse* and *Psychological Neglect*. The scale is scored on a 5-point scale, with 0 = none, 1 = rarely, 2 = sometimes, 3 = often, and 4 = always. Higher CPANS scores indicate more severe psychological abuse/neglect experienced during childhood. In this sample, the Cronbach’s alpha was 0.941.

#### Sleep problems

The Pittsburgh Sleep Quality Index (PSQI) was adopted to assess sleep problems in the past month [30]. The scale consists of 19 self-assessed and 5 other-assessed items, with the 19th self-assessed item and the 5th other-assessed item not participating in the scoring. The scale consists of seven components relating to sleep, i.e., quality, onset, duration, efficiency, disturbance, hypnotic medication, and daytime dysfunction, each of which is scored on a scale of 0–3. In this sample, the Cronbach’s alpha was 0.855.

#### Depressed mood

Depressed mood was measured using the Chinese version of Anxiety and Stress Scale − 21 Items (DASS-21) [31]. The 21-item scale includes three types of negative emotional experiences: depressed mood, anxiety and stress, with the depressed mood factor consisting of seven items. The scale is scored on a 4-point scale, with 0 = not at all, 1 = partially, 2 = mostly, and 3 = fully. The higher the total score on the depressed mood factor of the scale, the more intense the experience of depressed mood. In the present sample, the Cronbach’s alpha for the depressed mood factor was 0.844.

#### Suicidal ideation

Suicidal ideation in the past two weeks was assessed using the Chinese version of the Positive and Negative Suicide Ideation Inventory (PANSI) [32]. The scale consists of 14 items, divided into two dimensions: positive suicidal ideation and negative suicidal ideation. The scale is scored on a 5-point scale, 1 = never, 2 = rarely, 3 = sometimes, 4 = often and 5 = always. PANSI has two ways of evaluation. When screening for suicidal ideation, the threshold is set at a value ≥ 1.63 for the negative ideation scale and ≤ 3.33 for the positive one. When calculating the total suicidal ideation score, the inverse scoring was utilized for instances of positive suicidal ideation. A higher PANSI score indicates a higher level of suicidal ideation. In this sample, the Cronbach’s alpha was 0.881.

#### Covariates

In previous studies, a number of personal (age, gender, etc.) and family (parental marital status, etc.) correlates have been suggested to be associated with suicidal ideation [33, 34]. Based on the above findings, we selected these variables as possible covariates. Detailed information is given in Table 1.

### Statistical analysis

Initially, descriptive analyses were performed to examine the prevalence of psychological abuse/neglect, as well as suicidal ideation. For continuous variables, two independent samples t-tests were employed, while chi-square tests were utilized for group comparisons of categorical variables, in order to ascertain whether characteristics significantly differed between students with and without suicidal ideation (refer to Table 1). Spearman correlation analyses were executed on the primary variables (see Table 2). Subsequently, a path analysis was carried out to investigate the proposed mediating model. This analysis was implemented using Model 6 of the PROCESS macro program, as provided by Hayes. Significant variables identified in Table 1 were incorporated into the model, with psychological abuse/neglect as independent variables, suicidal ideation as the dependent variable, and sleep disturbances along with depressed mood functioning as chain mediators (refer to Fig. 1). To assess the significance of the mediating effects, a bias-corrected percentile Bootstrap method was employed. A total of 5,000 replicate samples were reinserted to obtain 95% confidence intervals for the mediating effects, and the mediating path was deemed significant if the aforementioned 95% confidence interval did not encompass 0. All data were analyzed utilizing IBM SPSS 25.0, and differences with two-tailed *p*-values < 0.05 were regarded as statistically significant.

## Results

### Common method deviation test

The Harman one-way test was used to test for common method bias. The results showed 10 factors with a characteristic root greater than one, of which the cumulative variance explained by the first factor was 28.7%. The value is less than 40%, indicating no serious problem of common method bias in this study.

### Descriptive analysis

We surveyed 14,210 students, well above the minimum sample size (see Methods), and eventually collected 12,192 valid questionnaires, corresponding to a response rate of 85.8%. The mean age of the survey respondents was 15.05 years (SD = 1.40) and 53.3% were boy. The term “left-behind experience” pertains to the situation of children aged 16 years or younger who have been left in their place of household registration for a duration of six months or more, while one or both of their parents sought employment elsewhere. Consequently, these children were unable to reside with both parents during this period [35]. The percentage of children left behind is 66.3%. In the present sample, 34.8% of students indicated experiences of psychological abuse/neglect, with girls demonstrating a higher likelihood than boys to report such incidents. Moreover, the overall prevalence of suicidal ideation among adolescents was approximated at 13%, with girl students exhibiting a heightened risk for suicidal ideation in comparison to their boy counterparts. Table 1 displays the characteristics of suicidal ideation. The results revealed that factors such as age, gender, left-behind experience, parental marital status, being an only child, and long-term medication use were associated with suicidal ideation. The median, interquartile spacing and correlation coefficients among the main study variables are shown in Table 2.

**Table 1:** Characteristics of suicidal ideation

Characteristics	Overall n(%)	Suicidal ideation		χ²/t	*p*-value
		No(%)	Yes(%)		
Age,M(SD)	15.05(1.40)	15.06(1.41)	14.97(1.28)	2.50	0.01
Gender				90.49	< 0.001
Boy	6502(53.3)	5831(55.0)	671(42.2)		
Girl	5690(46.7)	4772(45.0)	918(57.8)		
Ethnic group				0.33	0.57
Han ethnicity	12,113(99.4)	10,536(99.4)	1577(99.2)		
Ethnic minority	79(0.6)	67(0.6)	12(0.8)		
Left-behind experience				8.43	0.004
Yes	8087(66.3)	6982(65.8)	1105(69.5)		
No	4105(33.7)	3621(34.2)	484(30.5)		
Marital status of parents				52.34	< 0.001
Married	10,392(85.2)	9133(86.1)	1259(79.2)		
Divorced	1800(14.8)	1470(13.9)	330(20.8)		
Living environments				0.03	0.87
Urban area	3467(28.4)	3018(28.5)	449(28.3)		
villages	8725(71.6)	7585(71.5)	1140(71.7)		
Being the single child				13.14	< 0.001
Yes	1649(13.5)	1388(13.1)	261(16.4)		
No	10,543(86.5)	9215(86.9)	1328(83.6)		
Long-term medication use				125.41	< 0.001
Yes	264(2.2)	169(1.6)	95(6.0)		
No	11,928(97.8)	10,434(98.4)	1494(94.0)		

**Table 2:** Descriptive statistics and correlations for key variables

Variable	M	P25,P75	1	2	3	4
Psychological abuse/neglect	22	10,37	1.00			
Sleep problems	4	2,6	0.47***	1.00		
Depressed mood	9	7,12	0.57***	0.52***	1.00	
Suicidal ideation	24	20,31	0.45***	0.44***	0.57***	1.00

Note: ***p < 0.001

### Testing of the proposed model

Pathway analyses were conducted to investigate the mediating roles of sleep disturbances and depressed moods in the relationship between psychological abuse/neglect and suicidal ideation. These analyses were performed after accounting for potential confounding factors such as age, gender, experience of being left behind, parental marital status, being an only child, and long-term medication use.

Utilizing Bootstrap sampling to test for mediation effects, we found that (refer to Table 3) the indirect effect of the pathway involving sleep disturbances as the mediator was 0.07 (95% CI=[0.07, 0.08]), while that with depressive symptoms as the mediator was 0.20 (95% CI=[0.18, 0.21]). These results support hypotheses 1 and 2. Furthermore, the combined indirect effects of sleep disturbances and depressed moods as mediators were 0.07 (95% CI=[0.06, 0.08]). The total indirect effect of all pathways was 0.34 (95% CI=[0.33, 0.36]), meaning that, in the positive relationship between psychological abuse/neglect and suicidal ideation, there is a chain mediation involving sleep disturbances and depressive symptoms. This finding lends support to Hypothesis 3.

**Table 3:** Standardized path from the mediation analyses

	β	SE	*p*	BC 95% CI
**Direct effects from PA/N to SI**				
PA/N→SI	0.05	0.003	< 0.001	0.04,0.06
**Indirect effects from PA/N to SI**				
PA/N→SP→SI	0.07	0.01		0.07,0.08
PA/N→DM→SI	0.20	0.01		0.18,0.21
PA/N→SP→DM→SI	0.07	0.003		0.06,0.08
**Combined indirect effects**	0.34	0.01		0.33,0.36
**Effect of covariates**				
Age	-0.02	0.05	0.05	-0.18,0.0004
Gender	0.08	0.13	< 0.001	1.11,1.61
Experience of being left behind	0.004	0.14	0.65	-0.20,0.34
Marital status of parents	0.03	0.19	< 0.001	0.30,1.03
Being the single child or not	0.01	0.19	0.09	-0.05,0.71
Long-term medication use or not	0.07	0.44	< 0.001	2.96,4.70

Note: PA/N = Psychological abuse/neglect;SP = Sleep problems; DM = Depressed mood;SI = Suicidal ideation

Gender: 1=“boy”, 2=“girl”; Left-behind experience: 1=“yes”, 2=“no”; Marital status of parents: 1=“Not divorced”, 2=“Divorced”; Being the single children or not: 1=“No”, 2=“Yes”; Long-term medication use or not: 1=“No”, 2=“yes”

## Discussion

This study is the first to examine the roles of psychological abuse/neglect, sleep problems and depressed mood in the development of suicidal ideation among adolescents in western China, aiming to provide anticipatory guidance for individuals to improve their mental health. Our main findings include: (1) Experiences of psychological abuse/neglect, as well as suicidal ideation, are highly prevalent among Chinese adolescents; (2) psychological abuse/neglect, sleep disturbances, and depressed mood exhibit strong associations with suicidal ideation; (3) Sleep disturbances and depressed mood serve as mediators in the relationship between psychological abuse/neglect and adolescent suicidal ideation.

### Prevalence of suicidal ideation

Our survey found a 13% prevalence of suicidal ideation among adolescents, similar to previous results from Shandong and Fujian, China [36, 37]. We found a higher rate of suicidal ideation among girls than boys, which is consistent with the findings of a study that pooled and analysed suicidal behaviour among adolescents in 90 countries [38]. The observed gender disparity could be attributed to several factors. Adolescent girls may encounter heightened stress levels compared to boys, stemming from diverse aspects of physical maturation and scholastic adaptation. Additionally, research suggests that girls exhibit a higher susceptibility to psychological manifestations in response to stressors or traumatic events. Furthermore, cognitive traits such as increased propensity for worry and rumination are closely linked to anxiety and depressive disorders, which tend to be more prevalent among girls [39]. The prevalence of psychological abuse/neglect among females in our study was significantly higher than that among males. This might be another reason for the higher prevalence of suicidal ideation among girls compared to boys.

### The mediating role of sleep problems

The results of this study suggest that sleep problems are a mediator of the effect of psychological abuse/neglect on adolescent suicidal ideation, which supports Hypothesis 1. Specifically, adolescents who experienced psychological abuse/neglect had more severe sleep problems, which is consistent with previous research [40]. From a biological perspective, psychological abuse/neglect may activate the Hypothalamic-Pituitary-Adrenal (HPA) Axis [41]. Also, the HPA axis plays a role in sleep regulation and sleep disorders, and its activation can negatively affect sleep [42]. From a psychological perspective, good parenting/attachment styles are associated with good sleep [43], however, children who have experienced psychological abuse/neglect are less likely to have it. From a sociological perspective, parents who psychologically abuse and neglect their children are less likely to set bedtimes. Also, the children’s own inappropriate sleep habits may also be associated with increased sleep problems in their adolescents [44]. In addition, high levels of sleep problems are associated with high levels of suicidal ideation, which has been confirmed in previous studies [17]. 5-Hydroxytryptamine plays an important role in both suicide and sleep, and may mediate the relationship between sleep problems and suicidal ideation [45, 46]. Abnormalities in executive function due to nocturnal awakenings may also increase the risk of suicide [47].

### The mediating role of depressed mood

This study discovered that depressed mood served as an additional mediator between psychological abuse/neglect and suicidal ideation in adolescents, thus supporting Hypothesis 2. A positive correlation was observed, indicating that increased severity of psychological abuse/neglect was associated with heightened levels of depressed mood. These findings align with the outcomes of prior research in this domain [48]. The qualitative-stress model posits that mental illness or suicidal behavior arises from the interplay among an individual’s unique qualitative factors, cognitive vulnerability, and stressful life events [24]. Psychological abuse/neglect constitutes one such common and pervasive stressful life event, which can generate negative emotions in adolescents who have experienced it. Concurrently, depressed mood has been identified as the most critical direct predictor of suicidal ideation [49]. The mediating role of depressed mood in the relationship between stressful life events and suicidal ideation has been substantiated in prior research [50]. Furthermore, chronic stress is correlated with a hyperactive HPA axis and an elevated risk of depressed mood [51]. Previous studies have shown that the HPA axis stress response is a relatively stable risk factor for suicidal behaviour [52].

### Chain mediating effects of sleep problems and depressed mood

This study also discovered that psychological abuse/neglect influences depressed mood and, subsequently, suicidal ideation through sleep problems, corroborating Hypothesis 3. Specifically, heightened severity of psychological abuse/neglect results in greater sleep disturbances, which in turn lead to increased levels of depressed mood, ultimately elevating the risk of suicidal ideation. When individuals experience psychological abuse/neglect, they may repeatedly replay abuse-related words and images in their minds. Excessive rumination on the traumatic event can elicit distress, such as insomnia and nightmares, severely impacting sleep quality [53]. This compromised sleep quality may subsequently impair an individual’s emotional regulation capabilities [54], causing them to experience more negative emotions, which in turn increases the risk of suicide. The HPA axis may play an important role in this process [41, 42, 51, 52]. In addition, the chain mediated results of this study are consistent with the integrated motivational-volitional model of suicidal behaviour, in which, the development of suicidal behaviour undergoes three stages: the pre-motivational stage, the motivational stage and the volitional stage [55]. The first stage consists of quality-environment-life events, where qualities are biological, genetic, cognitive vulnerability factors or individual difference characteristics that increase the risk of suicide [55]. An example for this is sleep disorder. Negative life events experienced at any stage of life can pose a risk of suicide [56] e.g. psychological abuse/neglect. Both constitute triggering events and contextual factors for suicide. The motivational stage entails negative emotional experiences, with depressed mood generating feelings of distress, despair, and a desire to escape unbearable frustration and distress, potentially triggering suicidal ideation [57]. Lastly, the volitional stage involves the actualization of behavior, wherein suicidal ideation transforms into suicidal actions.

The study’s findings support the relationship between the pre-motivational and motivational stages. These results suggest that addressing sleep problems and depressed mood as two modifiable risk factors warrants attention in interventions and prevention efforts targeting suicidal ideation, particularly for adolescents who have experienced psychological abuse/neglect.

### Strengths and limitations

We employed a cluster sampling strategy that incorporated students from various schools, enabling us to obtain a large sample size. Nevertheless, as all participants in this study were adolescents from Sichuan Province, the generalizability of the findings may be constrained. Additionally, the self-reported nature of the data collection process may introduce recall bias into the current results. Furthermore, the cross-sectional design of this survey precludes the establishment of causal relationships between the study’s primary variables. Lastly, we did not distinctly differentiate psychological abuse from neglect experienced by the students, focusing solely on their cumulative effects. Consequently, only the relationship between their combined effects and other variables could be ascertained.

## Conclusion

13% of adolescents in this study have suicidal ideation; sleep problems and depressed mood play parallel and sequential mediating roles in the relationship between psychological abuse/neglect and suicidal ideation. Therefore, for adolescents who have experienced psychological abuse/neglect, developing good sleep habits and reducing depressed mood may help to reduce the risk of suicidal ideation. Future studies should employ both longitudinal and experimental designs to further examine the relationship between psychological abuse/neglect, suicidal ideation, sleep problem, and depressed mood.

## Data Availability

The datasets generated and/or analyzed during the current study are not publicly available but are available from the corresponding author on reasonable request.

## Abbreviations

PA/N: Psychological abuse/neglect
SP: Sleep problems
DM: Depressed mood
SI: Suicidal ideation
